# Lkb1 and Pten Synergise to Suppress mTOR-Mediated Tumorigenesis and Epithelial-Mesenchymal Transition in the Mouse Bladder

**DOI:** 10.1371/journal.pone.0016209

**Published:** 2011-01-19

**Authors:** Boris Y. Shorning, David Griffiths, Alan R. Clarke

**Affiliations:** 1 Cardiff School of Biosciences, Cardiff University, Cardiff, United Kingdom; 2 Department of Pathology, Cardiff University, Cardiff, United Kingdom; CNRS - Université Aix-Marseille, France

## Abstract

The AKT/PI3K/mTOR pathway is frequently altered in a range of human tumours, including bladder cancer. Here we report the phenotype of mice characterised by deletion of two key players in mTOR regulation, Pten and Lkb1, in a range of tissues including the mouse urothelium. Despite widespread recombination within the range of epithelial tissues, the primary phenotype we observe is the rapid onset of bladder tumorigenesis, with median onset of approximately 100 days. Single deletion of either Pten or Lkb1 had no effect on bladder cell proliferation or tumour formation. However, simultaneous deletion of Lkb1 and Pten led to an upregulation of the mTOR pathway and the hypoxia marker GLUT1, increased bladder epithelial cell proliferation and ultimately tumorigenesis. Bladder tissue also exhibited characteristic features of epithelial-mesenchymal transition, with loss of the epithelial markers E-cadherin and the tight junction protein ZO-1, and increases in the mesenchymal marker vimentin as well as nuclear localization of epithelial-mesenchymal transition (EMT) regulator Snail. We show that these effects were all dependent upon mTOR activity, as rapamycin treatment blocked both EMT and tumorigenesis. Our data therefore establish clear synergy between Lkb1 and Pten in controlling the mTOR pathway within bladder epithelium, and show that loss of this control leads to the disturbance of epithelial structure, EMT and ultimately tumorigenesis.

## Introduction

Deregulation of the PI3K pathway has been implicated in a range of human epithelial tumours. Both Pten and Lkb1 play fundamental roles in regulating this pathway, and mutations of both genes are associated with neoplasia. Mechanistically, Pten suppresses the PI3K pathway by dephosphorylating phosphatidylinositol-3, 4, 5-trisphosphates (PIP_3_) [1], [2]. PIP_3_ production causes Akt recruitment to the plasma membrane with subsequent Akt phosphorylation at Thr308 and Ser473 [3]. Phosphorylated Akt can then inhibit TSC2 with the subsequent activation of Rheb, mTOR, S6K p70 and stimulation of protein synthesis and cell growth [4]. Lkb1 also regulates mTOR via the TSC1/TSC2 complex as Lkb1 normally phosphorylates AMPK [5], for which TSC2 is a substrate [6]. Germline mutation of PTEN, LKB1 or TSC1/2 are all associated with human tumorigenesis (Cowden syndrome, Peutz-Jeghers syndrome and tuberous sclerosis respectively), and it is notable that all three syndromes share characteristic features, particularly an increased predisposition to hamartoma development [7].

Bladder cancer is one the most common neoplasias worldwide [8], being more prevalent amongst white males and people who have been exposed to cigarette smoke or industrial chemicals [9], [10]. Almost all bladder cancers originate in the bladder epithelium (urothelium). At the time of diagnosis, bladder carcinoma is often non-invasive (superficial bladder cancers) and the tumors are confined to the mucosa or submucosa: and only 20 percent of bladder cancer cases have signs of local invasion and 3 percent have distant spread) [11]. However, the disease is able to progress as has been documented by histological follow up of non-invasive bladder cancer patients [12].

Recently, it has been suggested that epithelial-mesenchymal transition (EMT) may be critical for the progression of cancer [13]. During EMT cells lose adherence and tight junctions, exhibit changes in their cytoskeletal architecture, and they can break through the basal membrane and become metastatic [14]. These characteristics are broadly similar to the features associated with cells undergoing EMT during normal development [13]. EMT can be induced by a variety of different pathways, including TGF-beta [15], Wnt [16], Notch [17], integrin [18] and also AKT/PI3K/mTOR. For example, transfection of a constitutively active form of the p110 catalytic subunit of PI3K has been reported to induce loss of ZO-1 from tight junctions [19]; and cell lines expressing constitutively active Akt show increased motility and invasion, loss of cell-cell adhesion and reduced expression of E-cadherin [20]. Furthermore, inhibition of mTOR by rapamycin has been shown to decrease the invasive behavior of cells, such as following TGF-β induced EMT [21].

One of the most important inducers of EMT and tumour metastasis is hypoxia [22] and there is growing evidence of a complex interplay between the AKT/PI3K/mTOR pathway and hypoxia [23]–[28]. For example, hypoxia suppresses energy-costly mRNA translation and protein synthesis via AMPK and TSC2-mediated mTOR inhibition [24] or other mechanisms [25], [26]. Correspondingly, TSC2 deletion abolishes hypoxia induced cell cycle arrest and enhances cell proliferation [24]. Hypoxia is well recognized to be capable of inducing EMT in cancer cells and this can be prevented by PI3K inhibition [23]. Furthermore, rapamycin treatment can coincidentally suppress mTOR signalling, HIF-1α expression and tumorigenesis in Lkb1−/+ tumours [28].

AKT/PI3K/mTOR pathway alterations are very frequent in bladder cancer, with 73% of human bladder tumours characterised by alterations in one of the major pathway components: TSC1, PI3K (PIK3CA) or PTEN [29]. More than half of all the bladder tumours show loss of an allele of TSC1 [30]. PTEN expression has been reported to be reduced in 49% of tumours, and 25% are reported to be characterised by mutation of PIK3CA [29]. AKT1 mutations also can be involved in bladder cancer development [31] as well as germ line genetic variants in RAPTOR [32]. Furthermore, these pathway mutations are often not mutually exclusive, suggesting that they can have synergistic or additive effects upon tumorigenesis [29].

Although loss of heterozygosity at the PTEN locus is a very common event in muscle-invasive transitional cell carcinomas of the bladder [33], previous attempts to generate mouse models based upon Pten deficiency have shown rather mild phenotypes, varying from an incidence of 10% of transitional cell carcinomas [34] to no tumorigenesis at all [35], [36]. However, combinatorial mutation of both mouse Pten and p53 has been reported to result in invasive neoplasia [36].

These observations suggest that single mutations of the AKT/PI3K/mTOR pathway may be insufficient to drive bladder tumorigenesis, reflecting a capacity of the pathway to compensate for such single mutations. Lkb1 kinase is a tumour suppressor which regulates the TSC1/2 complex [6], which is often deregulated in human bladder cancer [29]. Thus, although Lkb1 is not been reported to be frequently mutated in this disease [29], it is clear that pathways governed by Lkb1 are relevant to disease and we therefore decided to evaluate the extent of in vivo synergy between mutations in Pten and Lkb1 within murine epithelia.

To achieve this, we analysed the phenotypes of loss of either or both Lkb1 and Pten from a range of mouse epithelia using a conditional AhCre-LoxP strategy. The overriding phenotype of these mice was of rapid onset of bladder EMT and neoplasia, both of which we show to be dependent upon MTOR activity. Our data therefore establish tissue specific synergy for these mutations in both EMT and ultimately tumorigenesis.

## Materials and Methods

### Ethics Statement

All animal work was performed in accordance with the UK Animal Scientific Procedures Act 1986 and was covered by both Project and Personal licences that were issued by the Home Office and reviewed by the Cardiff University Research Ethics Committee. Professor Alan R Clarke Project Licence numbers 30-2246 (2005–2010) and 30/2737 (years 2010–2015), as well as Dr Boris Shorning Personal Licence 60/8372 covered all procedures and breeding.

### Experimental Animals and Cre recombinase Induction

All experiments were conducted according to U.K. Home Office regulations. Mice were maintained on C57BL6/J-129/Ola-C3H outbred background and all mice were genotyped by PCR using DNA extracted with Puregene DNA extraction kit (Gentra systems), the primers were as described for the targeted Rosa26R [37], Lkb1^fl/fl^ [38], Pten^ fl/fl^ [39] and AhCreER™ transgene [40]. Cre recombinase activity was induced in all mice (both in AhCreER™ -negative and AhCreER™ -positive) by 4 intraperitoneal injections of 80 mg/kg beta-naphthoflavone combined together with 80 mg/kg of Tamoxifen (both were dissolved in corn oil). The mice received 4 injections in 2 days with a 12 hour span between each injection. All times indicated in the text were related to the time elapsed since the first exposure. Mice were sacrificed and dissected at a certain timepoint or to avoid unnecessary suffering. Their bladders were removed and measured with a fine calliper.

### β-Galactosidase analysis

Mouse bladder samples from 14 day induced animals were opened out, rinsed with cold water and fixed with pins on a wax plate. Following a quick fix in 2% formaldehyde/PBS/0.1% glutaraldehyde, bladders were demucified and left to stain in X-gal solution overnight as described for the intestinal samples in [36]. The recombination was assessed by blue staining in the tissue [40], [41].

### Western analysis

Bladder epithelium (from animals 50 days post induction) was peeled off the bladder wall, homogenized and solubilised in 500 µl of lysis buffer (50 mM Tris pH 7.5, 100 mM NaCl, 5 mM EDTA, 5 mM EGTA, 0.5% NP-40, 40 mM beta-glycerolphosphate, 0.5 µg/ml each Leupeptin, Pepstatin, Chymostatin, 50 mM NaF, 5 mM Na_3_VO_4_, with 1 µM microcystin) for 10–20 minutes on ice. Supernatants were aliquoted and snap frozen in liquid nitrogen. Protein concentrations were determined using a Coomassie based method (Bio-Rad). Equal amounts of cellular protein (20 µg) were separated on 10% acrylamide gel and subsequently transferred on Hybond ECL nitrocellulose membrane (Amersham Biosciences). Total protein was visualized with Ponceau (Sigma). After blocking the membranes in TBS containing 5% BSA, 0.05% Tween 20, and 0.02% NaN_3_ for 1 hour, primary antibodies were added in block solution for overnight incubation at 4°C. After five repeated five minute washes in TBS, 0.05% Tween 20, the appropriate HRP-conjugated secondary donkey antibodies (Amersham Biosciences) were added (dilution 1∶5000) for 30 minutes. After five washes (five minutes each) antibody binding was detected using ECL reagent (Amersham Biosciences). The sources and dilutions of the primary antibodies used for western blotting analysis are mouse monoclonal Lkb1, 1∶1000, from Upstate (clone5c10), rabbit polyclonal Pten, phospho-AMPK^T172^, phospho-mTOR^S2448^, phospho-S6^ S240/244^, phospho-IRS^S302^, 1∶1000, all from Cell Signalling, mouse monoclonal beta-actin, 1∶5000, from Sigma, rabbit polyclonal Raptor^Ser722^, 1∶500, from Millipore.

### Immunohistochemistry and immunofluorescence

Immunohistochemistry and immunofluorescence were performed on formalin fixed tissue. Bladders were opened, rinsed with water, and fixed without inflation in ice-cold 10% neutral buffered formalin for a maximum of 14 hours at 4°C. All fixed samples were embedded in paraffin and sectioned to 5–6 µm on poly L-lysine slides. Antigen retrieval was performed by boiling in citrate buffer (LabVision) for 10 minutes at 100°C. For the immunohistochemical procedure we blocked endogenous peroxidase by EnVision blocking solution (DakoCytomation) for 5 minutes. The non-specific binding was blocked for 30 minutes with either 5% goat serum (DakoCytomation) for the primary rabbit polyclonal antibodies or 5% rabbit serum (DakoCytomation) for the primary mouse monoclonal antibodies and primary antibody incubation was performed at 4°C overnight. The immunohistochemical detection was performed using secondary antibody horseradish peroxidase-labelled polymer and DAB reagent (DakoCytomation). Immunofluorescent detection was performed by anti-rabbit AlexaFluor 488 and anti-mouse AlexaFluor 594 secondary antibodies (Invitrogen). The sources and dilutions of the primary antibodies used for immunohistochemical analysis were: mouse monoclonal Ki67, 1∶1000 (Vector Laboratories), rabbit polyclonal phospho-AKT^Ser473^(1∶50), rabbit polyclonal IHC-specific phospho- mTOR^Ser 2448^(1∶100), phospho- ribosomal S6 protein ^Ser 240/244^ (1∶100), Caspase-3 (1∶100) all four from Cell Signalling, rabbit polyclonal GLUT1 (1∶250) and rabbit polyclonal Snail (1∶250) from Abcam (1∶250), mouse monoclonal E-cadherin (1∶100) from BD Biosciences, rabbit polyclonal ZO-1 (1∶50) from Zymed (1∶50), rabbit polyclonal Vimentin (1∶250) and goat phospho-Smad 2/3^Ser423/425^ (1∶500) from Santa Cruz.

## Results

### Lkb1 and Pten deletion from the mouse urothelium

**Figure 1:**
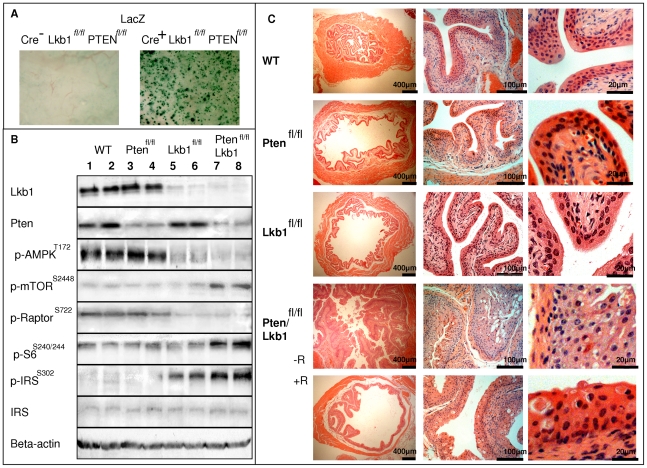
Lkb1 and Pten loss in the epithelium of mouse bladder after AhERCre recombinase induction. **A**, Cre-mediated recombination is marked by beta-galactosidase reporter gene expression in mouse bladder. AhERCre-positive bladder tissue (Pten -deficient) shows extensive recombination on day 14 after a regime of beta-naphtoflavone injections compared to no recombination in Cre-negative bladder tissue. **B**, Western blot detection of Lkb1, Pten, phospho-AMPK (Thr172), phospho-mTOR (S2448), phospho-S6 ribosomal protein (S240/244), IRS-1 and phospho-IRS-1 (S307) expression in induced Cre-negative *Lkb1^fl/fl^ Pten^ fl/fl^* (1, 2) and induced Cre-positive: *Pten^ fl/fl^* bladder epithelial tissue (3,4), *Lkb1^fl/fl^* bladder epithelial tissue (5,6), *Lkb1/Pten^ fl/fl^* bladder epithelial tissue (7, 8) at day 50. **C,** Hematoxylin and eosin staining of bladder sections of Cre-negative (WT) and recombined *Cre^+^ Pten^ fl/fl^*, *Cre^+^Lkb1^f/fl^*, *Cre^+^ Pten^ fl/fl^Lkb1^fl/fl^* mice (both treated and non-treated with rapamycin) at day 100 following a combined injection with beta-naphthoflavone and tamoxifen. Lkb1/Pten-deficient bladders show abnormal growth of urothelium which is suppressed by rapamycin. Scale bars correspond to 400 µm (left column), 100 µm (middle column) and 20 µm (right column).

We crossed mice bearing an inducible *AhCreER™* transgene [40] with mice bearing a LoxP flanked *Lkb1* cDNA cassette [38]; and with mice carrying a LoxP flanked *Pten* locus (exons 4 and 5, [39]). The *AhCreER™* transgene is conditional, being induced by exposure to beta-napthoflavone. Activity of the transgene is also tamoxifen dependent, as the Cre recombinase protein is fused with an estrogen receptor element [40]. This strategy drives extensive recombination in the small intestine, liver and bladder with lesser efficiency in stomach and gallbladder [40]. We generated four cohorts of mice, namely (a) *AhCreER^+^Lkb1^fl/fl^*, (b) *AhCreER^+^Pten^fl/fl^*, (c) *AhCreER^+^Lkb1^fl/fl^Pten^ fl/fl^* and (d) *AhCreER-*negative *Lkb1^fl/fl^Pten^fl/fl^* mice as a control cohort. Six week old mice from all four cohorts were given a series of intraperitoneal injections of beta-napthoflavone combined with tamoxifen (four injections in two days, 12 hours between each of the two injections). Beta-naphthoflavone induced Cre recombinase expression, and tamoxifen induced the translocation of Cre into the nucleus [40]. To determine the extent of recombination, we also introduced the LacZ Rosa reporter allele into a subset of animals [37]. As we have previously shown, this protocol resulted in high efficiency recombination in the range of mouse epithelia [40]. However, given the primary bladder phenotype (see below) we also now characterised recombination levels within bladder urothelium, which revealed extensive recombination in all cohorts (Fig. 1, A). To confirm gene deletion and reduced protein expression within bladder urothelium, we analysed levels of Lkb1 and Pten by western blot 50 days following induction. *AhCreER-*negative *Lkb1^fl/fl^Pten^fl/fl^* (WT mice) showed Lkb1 and Pten proteins present (Fig. 1, B, lanes 1,2), *AhCreER^+^Pten^fl/fl^* (Pten-deficient mice) showed reduced Pten protein levels (Fig. 1, B, lanes 3,4), *AhCreER^+^Lkb1^fl/fl^*(Lkb1-deficient mice) showed decreased Lkb1 protein levels (Fig. 1, B, lanes 5,6) and *AhCreER^+^Lkb1^fl/fl^Pten^ fl/fl^* (Lkb1/Pten-deficient mice) had reduced Lkb1 and Pten levels (Fig. 1, B, lanes 7,8).

### Combined deletion of Lkb1 and Pten in the mouse urothelium changes its morphology and proliferation levels and promotes tumorigenesis

Twenty-five mice from each cohort group were aged to investigate tumour incidence and survival. To further investigate the disease process, we also examined bladder phenotypes in a minimum of three animals from each genotype at days 50, 100 and 125.

Examination of bladder tissue revealed that Pten-deficient bladders and Lkb1-deficient bladders did not show any significant morphological differences from WT counterparts at day 50 (Fig. S1) as well as at day 100 (Fig. 1, C). In contrast, Lkb1/Pten-deficient mice showed a dramatic change in urothelial morphology, epithelium appeared thicker, with more mitotic cells both at day 50 (Fig. S1) and 100 (Fig. 1, C). At day 50 thickness of urothelium was 17.5±1.8 µm for WT, 19.5±1.6 µm for Pten-deficient, 16.0±2.1 µm for Lkb1-deficient and 58.5±5.4 µm for Lkb1/Pten-deficient urothelium. We did not observe urothelial hyperplasia detected at a similar timepoint (8 weeks) in Pten-deficient urothelium described earlier [34] and this discrepancy may be due to some differences in the genetic background of the experimental mice. Bladder epithelium with Lkb1/Pten double deletion also exhibited signs of impaired survival as assessed by the presence of apoptotic bodies and vacuoles containing cellular debris both at day 50 (Fig. S1) and day 100 (Fig. 1, C). We also performed Caspase-3 staining at day 100 to confirm that these are true apoptotic cells (Fig. S2).

**Figure 2:**
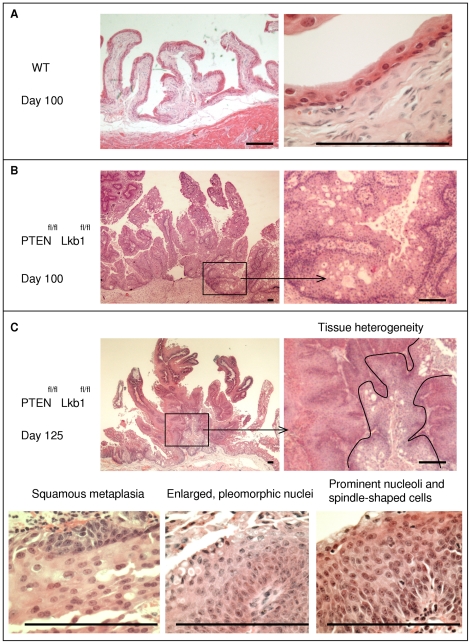
Bladder tumorigenesis after combined Lkb1 and Pten deletion. **A-C**, Hematoxylin and eosin staining of bladder sections of Cre-negative (**A**) and bladder tumour in recombined *Cre^+^ Pten^ fl/fl^Lkb1^fl/fl^* mice at day 100 (**B**) and day 125 (**C**) following combined injection with beta-naphthoflavone and tamoxifen. Borderlines show different areas of the tumour: more proliferative section is to the left and less proliferative (containing vesicles) is inside the marked area. Scale bars correspond to 100 µm.

By day 100 the mucosa of Lkb1/Pten -deficient bladders appeared thickened, the urothelium was diffusely hyperplasic and showed focal papillary growths with fine branching fibro-vascular cores and atypical epithelium 5 to 12 cells thick.(Fig. 1, C, Fig. 2, B).

By day 125, the cohort developed large papillary tumours which showed some tissue heterogeneity: it had tissue with vacuoles and apoptotic cells as well as much denser tissue lacking them (Fig. 2, C). This dense tissue showed no signs of apoptosis according to Caspase-3 staining suggesting that the survival disadvantage is not longer present in Lkb1/Pten-deficient bladder (Fig. S2). The tissue showed signs of spindle-shaped cells, squamous metaplasia, focal microvesicular change and a marked increase in nuclear-cytoplasmic ratio (Fig. 2, C). Nuclei were enlarged, pleomorphic and contained prominent nucleoli (Fig. 2, C). Neither day 100 nor day 125 tumours showed evidence of invasion of the lamina propria. Lkb1-deficient and Pten-deficient mice did not develop bladder tumours in the long term. Lkb1-deficient mice developed gastric polyps similar to those previously described [42], the onset of which was 270 days after induction Mice were killed shortly after this point and no bladder tumours were observed in these animals. Pten-deficient mice also did not develop bladder tumours at all the available timepoints (up to day 700).

**Figure 3:**
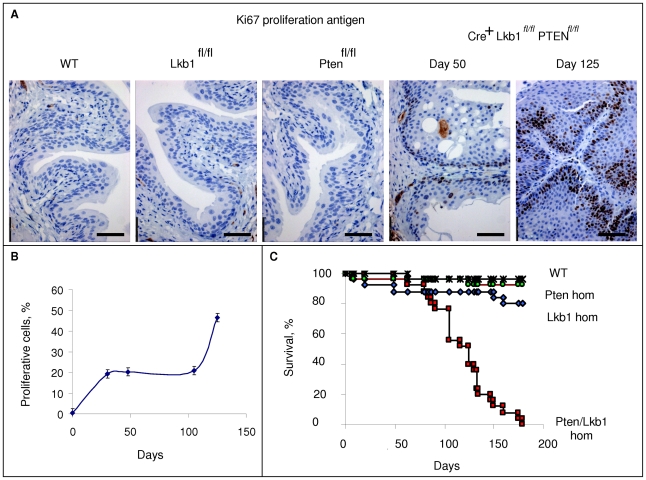
The combined loss of PTEN and Lkb1 induces bladder epithelium hyperproliferation and affects longevity. **A**, Immunohistochemical staining with anti-Ki67 antibody showing proliferative cells in the bladder epithelium of Cre-negative and recombined *Cre^+^Pten^fl/fl^*, *Cre^+^Lkb1^f/fl^*, *Cre^+^ Pten^ fl/fl^Lkb1^fl/fl^* mice (day 50 for all four genotypes and both days 50 and 125 for double-negatives) **B**, A graph showing the increase in proliferative cells in the bladder epithelium of *Cre^+^ Pten^ fl/fl^Lkb1^fl/fl^* mice, **C**, Kaplan-Meier plot of WT (*Cre^−^ Pten^fl/fl^Lkb1^fl/fl^)* cohort is represented in black stars (n = 25), *Cre^+^ Pten^ fl/fl^* cohort is represented in green (n = 25), *Cre^+^Lkb1^f/fl^*cohort is represented in blue (n = 25), *Cre^+^ Pten^fl/fl^Lkb1^fl/fl^*cohort represented in red (n = 25. *Cre^+^Pten^fl/fl^Lkb1^fl/fl^* mice show decreased longevity, where 100% of the cohort did not survive past day 179, χ^2^ tests confirmed that *Cre^+^Pten^fl/fl^Lkb1^fl/fl^* mice exhibit a significantly reduced average survival compared with Cre-negative cohort (P<0.0001, χ^2^ = 44.6), while other two cohorts do not show the same tendency: *Cre^+^Pten^fl/fl^* (P = 0.556, χ^2^ = 0.347), and *Cre^+^Lkb1^fl/fl^* (P = 0.083506 χ^2^ = 3.00). Scale bars correspond to 20 µm.

After day 100 we noticed a marked decrease in Lkb1/Pten-deficient cohort survival compared to WT, Lkb1-deficient and Pten-deficient cohorts. The majority of Lkb1/Pten-deficient animals became ill due to bladder blockage and they were therefore culled during the following 50 days (Fig. 3, C). Bladders from Lkb1-deficient and Pten-deficient cohorts of mice were not different in size from the Cre-negative cohort but Lkb1/Pten- deficient cohort bladders were enlarged, being 15.3±3.6 mm diameter by day 125 post-induction (the size of WT mouse bladder is 4.8±0.7 mm).

Urothelium with only Lkb1 deletion or only Pten deletion did not show any changes in proliferation compared to WT according to Ki67 staining (Fig. 3, A). WT urothelium had 0.38±0.07% Ki67-positive cells while Lkb1-deficient and Pten-deficient have 0.41±0.05% and 0.40±0.08%. By contrast, the deletion of both Lkb1 and Pten led to an increase in proliferation levels. Before induction, Lkb1/Pten^fl/fl^ bladders had 0.36±0.04% Ki67-positive cells but this figure rose to 19.39±3.3% by day 30 according to Ki67 staining (Fig. 3, A, B). The level of proliferation remained relatively stable until day 100 (20.98±3.1%), after which time a further elevation was observed, reaching 46.43±5.1% by day 125 (Fig. 3, B). After day 100, morphologically new structures were identifiable in the urothelium: these were highly proliferative areas with increased nucleus to cytoplasm ratio and they did not contain vesicles with cellular debris typical of the initial stage of tumorigenesis (Fig. 3, A).

As previously reported [40], the Ah-CreER™ transgene used here also drives recombination in other tissues, including stomach and small intestinal epithelium. In this study, the primary phenotype was observed in the bladder, and indeed all animals had to be killed as a direct consequence of bladder tumorigenesis and obstructed flow of urine. Our current data therefore indicates a particularly strong synergy between Lkb1 and Pten in this epithelial cell type.

### Lkb1 and Pten show cooperative action in control of the mTOR pathway in mouse urothelium

**Figure 4:**
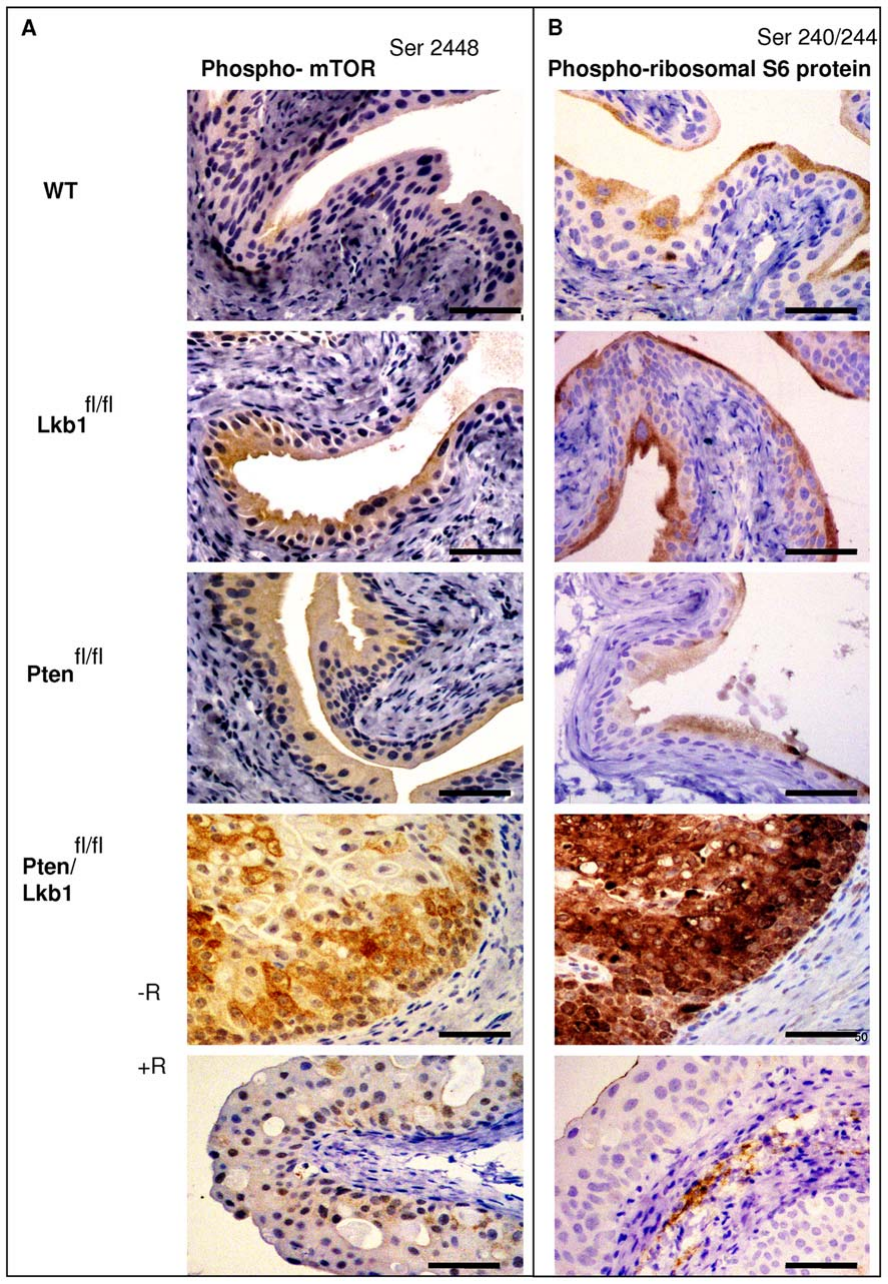
mTOR/S6K pathway deregulation in Lkb1/Pten-deficient mouse urothelium. The combined deletion of Lkb1 and Pten in the mouse urothelium led to drastic changes in mTOR/S6K pathway by day 100 after the induction and this is prevented by rapamycin treatment. **A**, **B**- Phospho- mTOR^Ser2448^ (**A**) and Phospho- ribosomal S6 protein^Ser240/244^ (**B**) immunostaining displaying elevated levels of mTOR and phosho-S6 ribosomal protein phosphorylation, Scale bars correspond to 50 µm.

Combined deletion of Lkb1 and Pten led to a significant increase in levels of phosphorylated mTOR^S2448^ and S6 ribosomal protein^S240/244^ according to western blot (Fig. 1, B). Immunohistochemical staining showed that the increase in mTOR^S2448^ and pS6^S40/244^ ribosomal protein (Fig. 4) occurred only in the epithelial layer of the mouse bladder of Lkb1/Pten-deficient mice. At the same time, urothelium deficient in only Lkb1 or Pten did not differ from WT in either mTOR^S2448^ or pS6^S240/244^ levels on a western blot (Fig. 1, B) and mTOR^S2448^ levels were only marginally higher in Lkb1 and Pten single mutants according to immunohistochemistry (Fig. 4A). No change was observed for pS6^S240/244^ (Fig. 4B).

**Figure 5:**
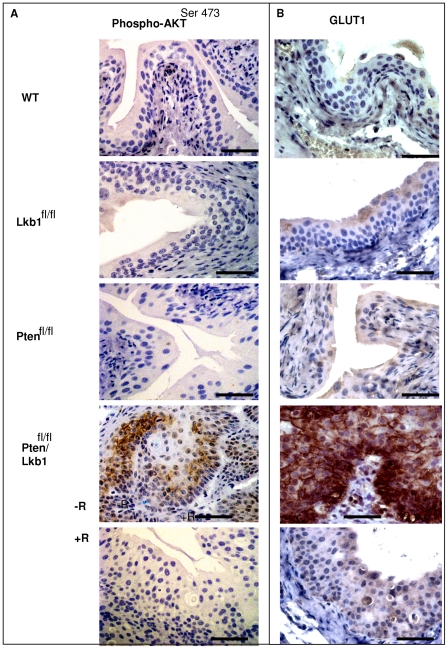
Increased Akt phosphorylation and hypoxia in Lkb1/Pten-deficient mouse urothelium. The drastic changes in AKT/mTOR/S6K pathway in Lkb1/Pten-double deficient urothelium corresponded to hypoxia marker GLUT1 upregulation by day 100 after the induction and this is prevented by rapamycin treatment. **A**, **B**- Immunostaining showing a strong increase in Phospho-AKT^Ser473^ (**A**) and GLUT1 (**B**) levels. Scale bars correspond to 50 µm.

Pten opposes the action of PI3K by dephosphorylating PIP3 and inhibiting the plasma membrane recruitment and phosphorylation of Akt [19]. We examined AKT phosphorylation on Ser473 as this strongly corresponds to Akt activation and occurs frequently in aggressive tumours [43]. AKT^Ser473^ levels were elevated in Lkb1/Pten-double negative urothelium (Fig. 5, A) in comparison with WT, Lkb1-deficient or Pten-deficient urothelium (we did not observe any difference in AKT^Ser473^ levels between these latter three cohorts).

It is known that Lkb1 mediates its inhibitory role on the mTOR pathway via phosphorylation of AMPK on Thr172 and subsequent TSC2 phosphorylation on T1227 and S1345 [6]. We therefore analysed levels of AMPK phosphorylation and found decreased AMPK^Thr172^ in both Lkb1-deficient and Pten/Lkb1-deficient mouse urothelia according western blot (Fig. 1, B). Levels of Ser^722^ phosphorylation of Raptor also reflected AMPK activation in accordance with a previous study [44]. Raptor was phosphorylated on this residue in WT and Pten-deficient samples and the band was much less prominent in the samples from Lkb1 and Pten/Lkb1-double deficient urothelium (Fig. 1, B). Unfortunately, anti-TSC2^T1227^ or anti-TSC2^S1345^ antibodies are not available and we not able to check the level of TSC2 phosphorylation on AMPK-dependent sites. TSC1 and TSC2 protein levels were not affected in all the four cohorts (data not shown).

Finally, we assessed the levels of S302 phosphorylation of IRS-1 as an indicator of the inhibitory feedback loop from mTOR/S6K which suppresses insulin signaling to PI3K [45]. This phosphorylation was the reason why insulin failed to activate AKT in TSC2-deficient cells [45]. We observed a slight increase in IRS-1^S302^ phosphorylation in Lkb1-deficient bladders and a strong increase in the double Lkb1/Pten bladder epithelium (Fig. 1, B). We did not observe a decrease in the overall IRS-1 expression (Fig. 1, B). We speculate that IRS-1 suppression in Lkb1-deficient urothelium may lead to a negative feedback suppression of Akt similarly to that observed in TSC2-deficient cells [45]. Wild type levels of activated Akt are low (Fig. 5, A) so it was not possible to assess its possible downregulation in Lkb1-deficient bladder.

Levels of mTOR phosphorylation in Lkb1-deficient urothelium appeared marginally higher than in WT by immunohistochemistry (Fig. 4, A). However, western blot detection was insufficiently sensitive to confirm any such increase (Fig. 1, B).

### The upregulation of mTOR pathway in mouse urothelium coincides with increased expression of the hypoxia marker GLUT1

Since AKT/PI3K/mTOR pathway regulation is implicated in hypoxia [23]–[28] we examined the expression of glucose transporter-1 protein (GLUT1), which has been reported to mark hypoxia in human bladder cancer [46] and functions as part of the glucose uptake machinery dependent on TSC2 phosphorylation and TSC inhibition of mTOR [47]. GLUT1 expression was strongly elevated in Lkb1/Pten -double negative urothelium in comparison with WT, Lkb1-deficient or Pten -deficient urothelium (Fig. 5, B). This protein is not normally expressed in the human bladder epithelium but it is present in malignant bladder and especially in muscle-invasive tumours when compared to superficial tumours [48].

### Rapamycin suppresses the effects of combined Pten and Lkb1 deletion in mouse urothelium

Since Lkb1 and Pten converge in mTOR control and given that deletion of these proteins drastically changes bladder tissue morphology we decided to confirm that the physiological effect of this simultaneous deletion was mediated by via mTOR. We therefore inhibited mTOR in the mouse bladder by exposure to rapamycin and determined the effect upon phenotype. For each genotype, ten animals were treated with vehicle and a further ten animals were treated with 1 mg/kg of rapamycin intraperitoneally every two days from day 50 onwards until day 100 when all the animals were sacrificed.

Tissue examination revealed that the bladder epithelium of rapamycin-treated WT, Pten -deficient and Lkb1-deficient animals showed no change in comparison to both vehicle-treated and untreated counterparts (data not shown). At the same time the regime of rapamycin injections had a substantial impact on Lkb1/Pten -deficient bladder. Rapamycin-treated Lkb1/Pten-deficient bladders were 5.1±1.2 mm in diameter whereas vehicle-treated were 14.6±4.1 mm. Rapamycin-treated Lkb1/Pten-deficient epithelium showed reduced thickness in comparison with non-treated, it had less mitotic cells whereas it retained some cells with apoptotic bodies and vacuoles (Fig. 1, C). Caspase-3 staining did not show an increase in apoptosis in rapamycin-treated Pten/Lkb1-deficient bladder compared to vehicle treated counterparts (Fig. S2). It therefore appears that the primary effect of rapamycin was to reduce mitosis levels. Apoptosis levels were relatively unaffected, although notably apoptosis levels were already very substantial in the Pten/Lkb1-double deficient normal urothelium (Fig. S2). The rapamycin-treated epithelium did not show hyperplasia or any papillary growths typical for vehicle-treated group (Fig. 1, C). Rapamycin treatment also strongly suppressed all the signs of mTOR hyperactivation in Lkb1/Pten-deficient bladders: phosphorylation of mTOR^S2448^ as well as AKT^Ser473^, and S6 ribosomal protein^S240/244^ (Fig. 4, 5). The expression of the hypoxic marker GLUT1 was also reduced, consistent with the direct involvement of mTOR in GLUT1 upregulation, and also with the observation of TSC2-mediated control of mTOR driven GLUT1 expression related to cell growth regulation [47]. Taken together, our data show that the effect of combined Lkb1 and Pten deletion are mediated via mTOR.

### mTOR activation in mouse bladder resulted in characteristic features of epithelial-mesenchymal transition (EMT)

**Figure 6:**
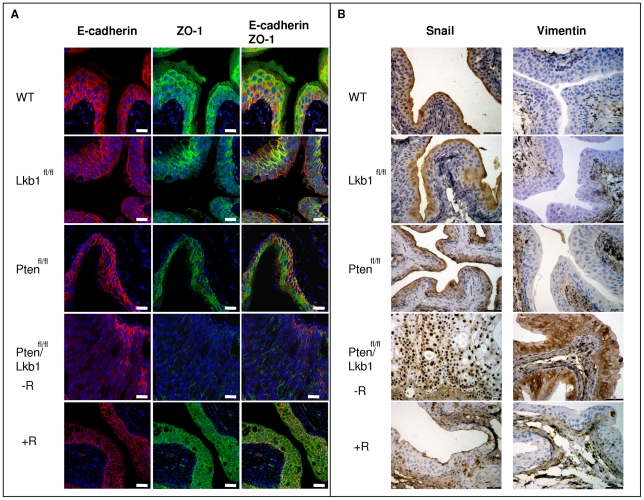
Lkb1/Pten-deficient mouse urothelium undergoes EMT. Mouse bladder epithelium deficient for both Lkb1 and Pten exhibits loss in epithelial markers and acquire mesenchymal properties by day 100. Rapamycin inhibition of mTOR suppresses the observed effect. **A**, E-cadherin and ZO-1 immunofluorescent co-localisation showing disappearance of these proteins from cell-cell walls in Lkb1/Pten-deficient bladder whereas rapamycin treatment prevents this loss, **B**, Immunostaining showing a strong increase in Vimentin levels and Snail nuclear localisation after the combined deletion of Lkb1 and Pten and this is mediated via mTOR evidenced from their suppressed levels in rapamycin-treated animals. Scale bars correspond to 20 µm.

The repression of both adherens and tight junction adhesion proteins is associated with EMT [49], [50]. Hence, expression levels of the cell adhesion marker E-cadherin correlates with stage, aggressiveness and progression of bladder cancer as well as survival [51]–[55]. Similarly, Zonula occludens 1 (ZO-1) is a tight junction marker which is lost during EMT induced by overactive PI3K [19]. To evaluate EMT within the bladder, we therefore performed immunofluorescent co-localization of both E-cadherin and ZO-1 in the mouse bladder and showed that in WT, Lkb1-deficient and Pten-deficient bladders they are both localised on the cell membrane, with ZO-1 showing a more punctate pattern marking tight junctions (Fig. 6, A). The simultaneous deletion of Lkb1 and Pten leads to a drastic decrease in the expression of these proteins whereas rapamycin treatment restores their expression (Fig. 6, A).

The expression of both E-cadherin and other EMT markers is controlled by Snail transcription factors and the ectopic expression of Snail in epithelial cells causes them to acquire tumorigenic and invasive properties [50]. High expression of Snail in human bladder tumours is linked to the higher risk of cancer recurrence [56]. We therefore analysed Snail expression in all our cohorts and found that Snail nuclear localisation drastically increased in Lkb1/Pten-double deficient epithelium and rapamycin treatment suppresses it (Fig. 6, B).

The observed disappearance of E-cadherin and ZO-1 from cell-cell junctions as well as the drastic rise in Snail nuclear localisation suggests that Lkb1/Pten-deficient tissue might have acquired mesenchymal properties. Vimentin is the most commonly used mesenchymal marker and its expression in epithelial tissues is normally associated with EMT [57]. Immunostaining showed that vimentin was not detectable in the urothelium of WT, Lkb1-deficient and Pten-deficient mice, whereas Lkb1/Pten-double deficient epithelium showed an increase in this marker (Fig. 6, B). Particularly high vimentin levels were observed in a subset of cells with spindle-shaped morphology typical for EMT (Fig. 6, B). Bladders from rapamycin-treated Lkb1/Pten-double deficient mice showed negligible vimentin staining similar to the other three genotypes (Fig. 6, B). Our data therefore argue that abnormally high mTOR signalling in Lkb1/Pten double deficient bladders results in a switch in urothelial cell differentiation towards a mesenchymal programme.

## Discussion

EMT represents the first step towards invasion and metastasis [12]. It is a complex mechanism that includes the remodeling of cell adhesion, disassembly of tight junctions including the loss of E-cadherin and ZO-1, and the transformation of polarized epithelial cells into an elongated fibroblastoid mesenchymal phenotype [12], [15]. EMT can be initiated by a spectrum of mutations or alterations in a range of signal transduction pathways and AKT/PI3K pathway has suggested to be at the centre in this mechanism [13], Here we report a new mouse model of bladder neoplasia with combined deletion of two genes controlling AKT/PI3K/mTOR pathway, Lkb1 and Pten in the urothelium (Fig. 1, A, B) and we show that this pathway has a crucial role in EMT occurring in this tissue (Fig. 6, A, B).

Pten deficiency is an important but not a sufficient factor in bladder cancer. Although Pten expression is reduced in almost half of human bladder cancer cases [29], only 10% of mice with urothelial deletion of Pten have been reported to develop papillary carcinomas [34]. Bladder tumours often have not just one but several alterations in the expression of several components of AKT/PI3K/mTOR pathway [29] suggesting the importance of synergistic effect of different tumour suppressors. We therefore hypothesized that tumorigenesis following loss of function of Pten in the bladder may be dependent on the activity of other mechanisms regulating mTOR. A very large proportion of bladder tumours lost an allele of TSC1 [30]. TSC1 forms a complex with TSC2 and its mutations were found in a number of bladder cancer cases as well [29]. We aimed to investigate Pten deficiency in the background of TSC1/2 downregulation. Although Lkb1 was not previously linked with bladder cancer and very few Lkb1 mutations were found in human bladder cancer [29], this kinase is a known regulator of TSC1/2 complex via AMPK and subsequent suppression of mTOR [6].

We tested whether Lkb1 deficiency alters tumorigenesis in a Pten-deficient setting. To achieve this, we created several cohorts of mice where either Lkb1 or Pten or both proteins were efficiently deleted from mouse urothelium (Fig. 1A, B). Cohorts with single Lkb1 or Pten deletions did not differ from WT but Lkb1/Pten double deficient urothelium exhibited drastic changes in tissue architecture (Fig. 1, C), tumorigenesis (Fig. 2) and proliferation (Fig. 3, A, B) as well as compromised animal survival (Fig. 3, C).

Lkb1/Pten deficient urothelium exhibited hyperactivation of mTOR/S6K pathway components: phospho-AKT^S473^, mTOR^S2448^, phospho-S6^S240/244^–ribosomal protein (Fig. 1, B, Fig. 4, 5). Rapamycin treatment was an effective tool to suppress this rise in mTOR signalling (Fig. 4) and it also suppressed other mTOR-driven events occurring in Lkb1/Pten deficient urothelium: EMT (Fig. 6, A, B) as well as hypoxia marker GLUT1 expression (Fig. 5B). We did not observe elevated levels of components of mTOR/S6K pathway in case of single Pten or Lkb1 deletion in the bladder, however we show that Lkb1 deletion led to increased Ser302 phosphorylation of IRS-1 (equivalent to human Ser307 residue) by p70S6K (Fig. 1, B). This suggests that, in the absence of Lkb1, there was at least partial activation of the feedback mechanism suppressing insulin signaling to PI3K which may account for the lack of any observed phenotypic change in the single mutants.

Why Lkb1 sporadic mutations are not common in human bladder cancer [29] is an outstanding question considering both our findings as well as its known function in regulating TSC1/2 complex [6]. It remains possible that Lkb1 is epigenetically repressed, although we are currently not aware of any reports supporting this. Alternatively, it may be possible that in the human bladder, loss of Lkb1 results in a cellular survival disadvantage due to increased sensitivity to metabolic stress, and that this precludes LKB1 driven tumorigenesis. Clearly, in the mouse urothelium any such disadvantage is insufficient to block tumorigenesis, although of note we did observe an increase in apoptosis in Lkb1/Pten-deficient urothelium (Fig. S2). In the light of this a double cross of Pten with Tsc1 (or Tsc2) may clarify the mechanism of bladder tumorigenesis.

Our observations correspond to the previous findings about AKT/PI3K/mTOR pathway involvement in EMT in other tissues and organs [12]–[14]. In our model the combined deletion of both Lkb1 and Pten in the mouse bladder drastically activates mTOR pathway and this precise activation serves as a signal for EMT program execution (Fig. 6). When mTOR was suppressed with rapamycin injections, bladders showed no signs of EMT (Fig. 6). Lkb1/Pten doubly deficient bladder exhibited loss of both adherence (E-cadherin) and tight junction (ZO-1) markers (Fig. 6, A), together with elevation of the nuclear localization of Snail transcription factor and increase in Vimentin mesenchymal marker (Fig. 6, B). The observed phenotype also included elevation of the hypoxia marker GLUT1 and this also was due to deregulated mTOR since rapamycin injections prevented GLUT1 upregulation (Fig. 5, B). Tumour hypoxia is usually a sign of poor prognosis and resistance to radiation therapy [57] and hypoxic conditions can be a prerequisite to EMT and consequently can lead to invasion metastasis [58], [ 59]. We did not observe invasion in our model but we realize that our time frame could be insufficient; with tumours developing rapidly.

The inhibition of EMT by rapamycin (Fig. 6) argues that the bladder phenotype is primarily due to elevated mTOR activity; however it has been shown by others that EMT can be controlled by a complex variety of signal transduction pathways [15]–[18]. Clearly, it will be interesting to investigate possible cross-talk between mTOR and these other pathways in EMT induction, particularly with TGF-β since it cooperates with mTOR in EMT induction [21].

mTOR hyperactivation in the mouse bladder led to a combination of EMT and abnormal proliferation. Normal bladder epithelium is known to be a slowly proliferating tissue (unless injured), with the complete renewal of the urothelial cell population taking the whole lifespan of the mouse [60]. Cell proliferation in either Pten or Lkb1 deficient epithelia remained low as shown by Ki67 immunostaining, with no overt proliferative advantage following gene deletion (Fig. 3, A). The morphology in Pten and Lkb1-deficient epithelium was also not changed (Fig. 1, C). When Pten and Lkb1 were deleted in murine urothelium simultaneously, this invariably resulted in hyperproliferation and hyperplasia in all the animals from the Lkb1/Pten-deficient cohort. These effects on bladder growth were truly mediated by mTOR since rapamycin treatment prevented all the signs of hyperplasia. Rapamycin did not enhance apoptosis in Pten/Lkb1-deficient urothelium (Fig. S2) but acted as the suppressor of mTOR-mediated cell growth similarly to the urothelial model of Pten/p53 deficiency [36]. The simultaneous deletion of Lkb1 and Pten in the mouse bladder resulted in diffusely hyperplasic atypical urothelium with papillary growths and the tumours were histologically similar to human neoplasms: by day 100 resembled a low grade papillary urothelial carcinoma and turned into high grade by day 125 [61]. By day 125 the morphology of the tumours altered, such that they appeared more dense and proliferative (Fig. 2, C). This coincided with a decrease in animal survival due to bladder obstruction. Apart from the synergy in the bladder we also observed some effects of simultaneous deletion of Lkb1 and Pten on cell proliferation in the pyloric section of the stomach (data not shown). Small intestines of Lkb1/Pten-deficient mice did not however develop tumours despite extensive recombination in that tissue, with the epithelium showed a similar phenotype to that described previously for the single mutants [62], [63]. We therefore speculate that the mode of regulation of mTOR is tissue-specific and is perhaps more tightly regulated in highly proliferative tissues (such as the small intestine), as opposed to the less proliferative bladder.

We show here for the first time the phenotype of homozygous deletion of both Lkb1 and Pten in a given epithelium and demonstrate clear synergy in mTOR control in mouse urothelium, a synergy which normally leads to tumour suppression in this tissue. We conclude that altered mTOR leads to EMT and tumorigenesis in the mouse bladder. The data show the necessity of tight control of PI3K/AKT/mTOR pathway in the mouse bladder and also suggest that the mouse bladder can be a useful model for EMT-related research.

## Supporting Information

Figure S1 **Hematoxylin and eosin staining of bladder sections of Cre-negative and recombined** ***Cre^+^Pten^fl/fl^*** **,** ***Cre^+^Lkb1^f/fl^***, ***Cre^+^Pten^fl/fl^Lkb1^fl/fl^*** **mice at day 50 following a combined injection with beta-naphthoflavone and tamoxifen.** Scale bars correspond to 500 µm (left column) and 100 µm (right column).
(TIF)

Figure S2 **Lkb1/Pten-deficient mouse urothelium exhibits Caspase-3 elevation up to day 100 and with zones devoid of apoptosis appearing at the later stages (day 125).** Rapamycin inhibition of mTOR suppresses apoptosis. Scale bars correspond to 50 µm.
(TIF)
