# Supplementary figures and images for: Lkb1 and Pten Synergise to Suppress mTOR-Mediated Tumorigenesis and Epithelial-Mesenchymal Transition in the Mouse Bladder
Source: PLoS One. 2011 Jan 19;6(1):e16209. doi: 10.1371/journal.pone.0016209 (PMC3023771; doi:10.1371/journal.pone.0016209)

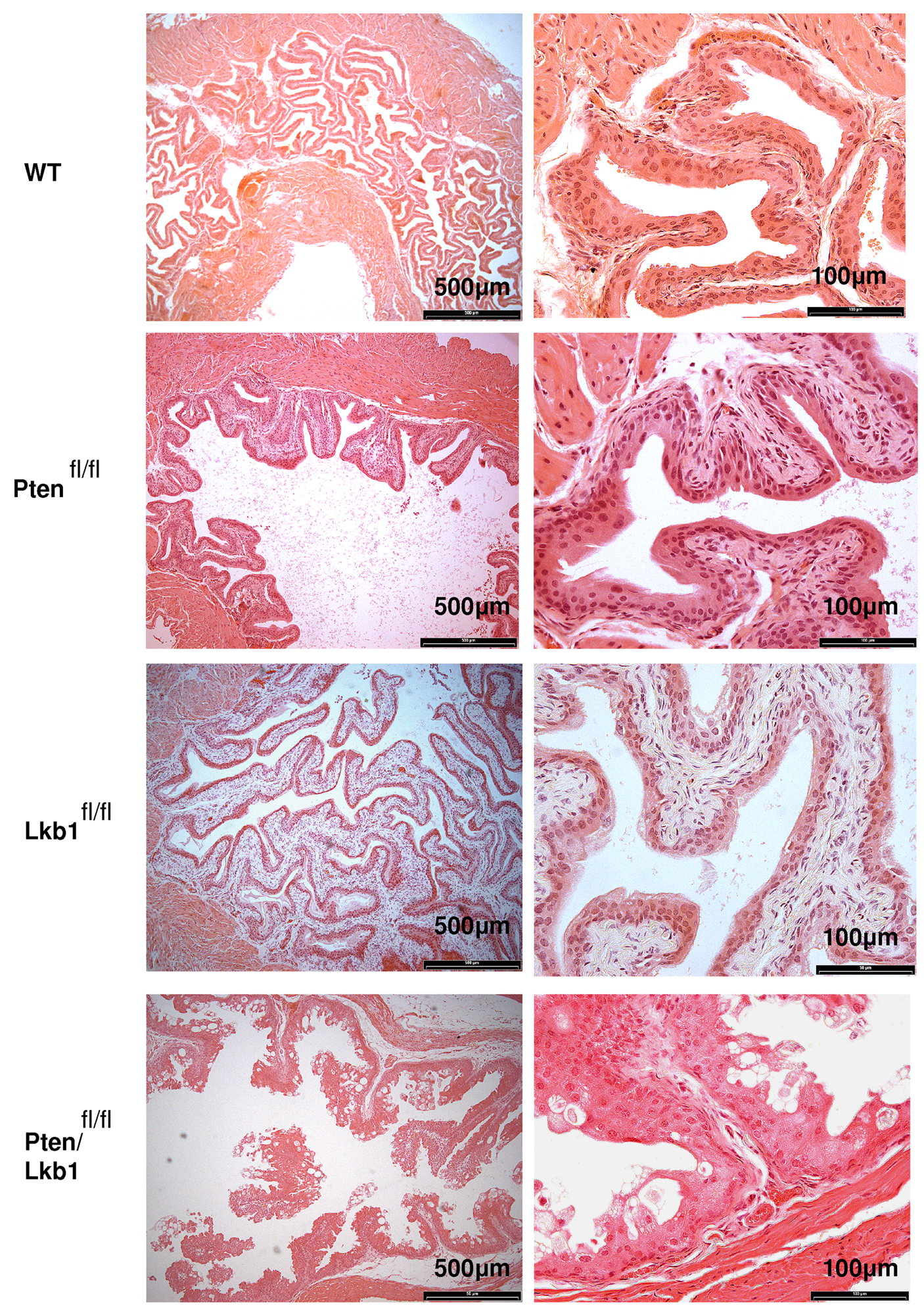

Supplement: Figure S1 — Hematoxylin and eosin staining of bladder sections of Cre-negative and recombined Cre+Ptenfl/fl , Cre+Lkb1f/fl, Cre+Ptenfl/flLkb1fl/fl mice at day 50 following a combined injection with beta-naphthoflavone and tamoxifen. Scale bars correspond to 500 µm (left column) and 100 µm (right column). (TIF) [file pone.0016209.s001.tif]

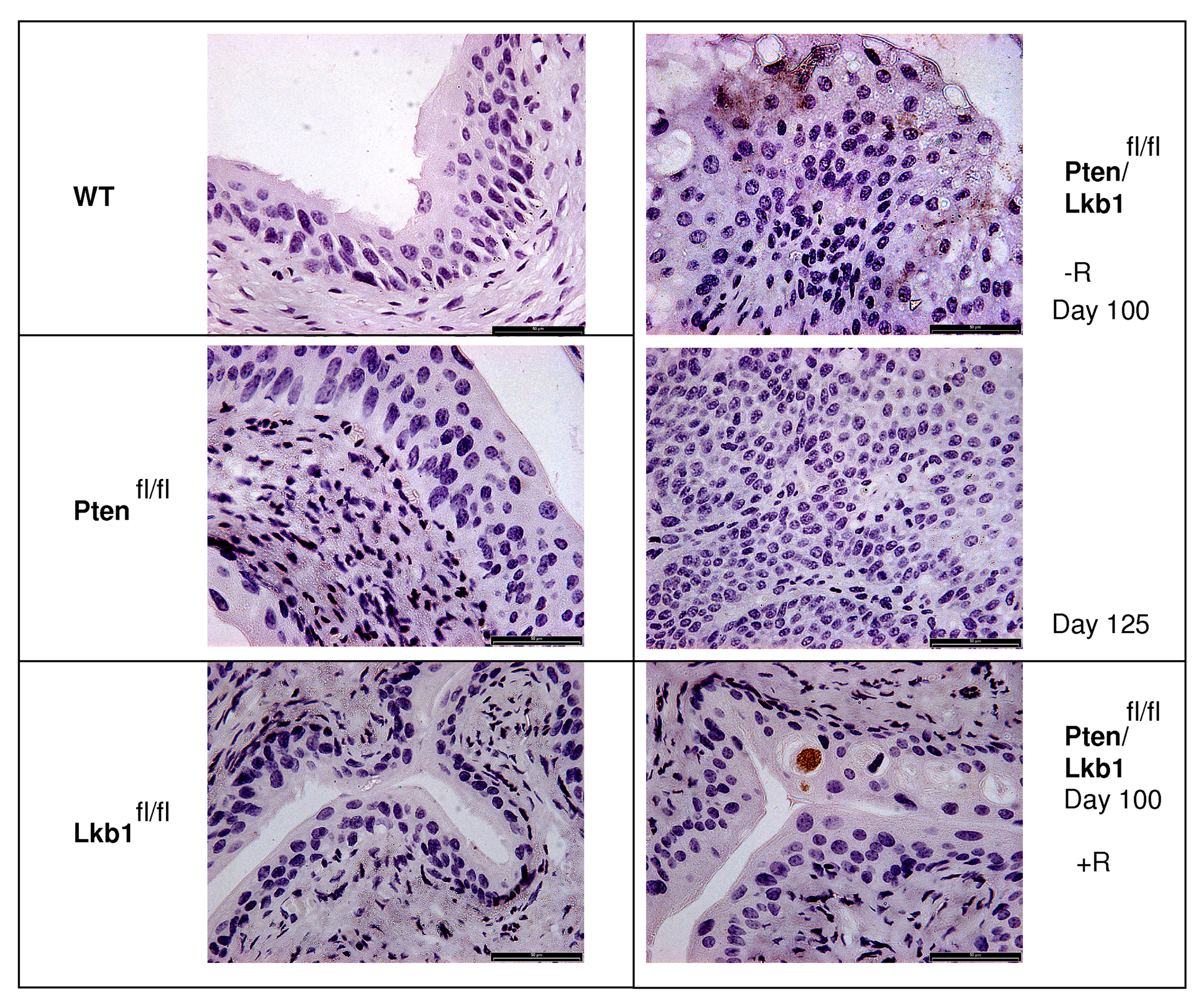

Supplement: Figure S2 — Lkb1/Pten-deficient mouse urothelium exhibits Caspase-3 elevation up to day 100 and with zones devoid of apoptosis appearing at the later stages (day 125). Rapamycin inhibition of mTOR suppresses apoptosis. Scale bars correspond to 50 µm. (TIF) [file pone.0016209.s002.tif]
